# Editorial: Fermented foods in modern nutrition: exploring health benefits and research innovations

**DOI:** 10.3389/fnut.2026.1890667

**Published:** 2026-06-18

**Authors:** Ragini Bodade, Tanaji Kudre, Morena Gabriele, Gabriel Cardoso-Ugarte, Fatih Ozogul, Dorota Zielinska, Anna Łepecka

**Affiliations:** 1 Life Science Division, Institute of Advanced Study in Science and Technology, Guwahati, Assam, India; 2 Academy of Scientific and Innovative Research (AcSIR), Ghaziabad, Uttar Pradesh, India; 3 Council of Scientific & Industrial Research-Central Food Technological Research Institute, Mysore, Karnataka, India; 4 National Research Council, Institute of Agricultural Biology and Biotechnology (CNR-IBBA), Pisa, Italy; 5 Popular Autonomous University of the State of Puebla, Puebla, Mexico; 6 Department of Seafood Processing Technology, Faculty of Fisheries, Cukurova University, Adana, Türkiye; 7 Warsaw University of Life Sciences, Warsaw, Poland; 8 Institute of Agricultural and Food Biotechnology—State Research Institute, Warsaw, Poland

**Keywords:** fermented foods, food preservation, health benefits, lactic acid bacteria, metabolic diseases, prebiotics, probiotics

Fermented foods and probiotic-based interventions have emerged as important modulators of gut microbiota, metabolic homeostasis, and chronic disease management. This Research Topic brings 11 articles, including original research articles and review articles, that collectively highlight recent advances in fermented food biotechnology, probiotic functionality, microbial ecology, and health-promoting bioactive compounds. Recent studies have demonstrated that metabolites derived from fermented foods, including short-chain fatty acid levels (SCFA), exopolysaccharides, organic acids, peptides, and phenolic derivatives, contribute significantly to gastrointestinal health, immune regulation, obesity management, and the prevention of metabolic disorders.

Regarding this, Lu et al. evaluated the therapeutic efficacy of fermented bamboo shoots in dextran sulfate sodium (DSS)-induced ulcerative colitis models. Fermented bamboo shoots administration significantly reduced the levels of pro-inflammatory cytokines TNF-α, IL-1β, and IL-6, elevated SCFA levels, and restored the populations of beneficial gut microbiota, including *Akkermansia, Anaerovorax*, and *Bacteroides*. These results suggest that fermented bamboo shoots may serve as an important dietary intervention in the management of inflammatory bowel disease. Likewise, Saleem et al. evaluated bacterial isolates from traditional fermented buffalo milk products and reported strong antioxidant and stress-adaptive properties in both lactic acid bacteria and non-lactic acid bacterial strains. The isolates showed tolerance to high NaCl concentrations, acidic pH, elevated temperatures, and bile salts, indicating their suitability for probiotic applications. *Lactobacillus brevis* Cc3 exhibited relatively higher antioxidant activity than *Streptococcus thermophilus* Y6, suggesting its possible role in oxidative stress alleviation and gastrointestinal health promotion in South Asian dietary systems.

The importance of fermented pseudocereals has also increased in recent years. Li et al. reviewed the development of fermented quinoa-derived products, including beverages, dairy alternatives, baked products, teas, and condiments. Their study highlighted the role of fermentation in improving nutrient bioavailability, sensory quality, shelf stability, and functional properties while addressing technological challenges associated with industrial-scale quinoa fermentation. The review further emphasized the need for process standardization and mechanized fermentation systems to support commercial production.

Agricultural by-products are also being explored as substrates for functional fermented foods. Jiang et al. demonstrated that broccoli stem by-products (BsBP) fermented using *Lacticaseibacillus casei* YC5 and *Pediococcus pentosaceus* RBHZ36 significantly alleviated obesity-associated metabolic disturbances in high-fat diet-induced murine models. Fermented BsBP further reduced body weight gain, adiposity, serum lipid accumulation, hepatic injury markers, and inflammatory cytokines. Additionally, gut microbial dysbiosis was restored through enrichment of beneficial taxa such as *Akkermansia* and *Bacteroides* and reduction of obesity-associated genera including *Colidextribacter, Helicobacter*, and *Mucispirillum*. Moreover, functional analysis is further associated with these microbial changes to improve energy metabolism and activation of the PPAR signaling pathway, confirming the therapeutic potential of fermented vegetable by-products as anti-obesity functional foods.

Fermented soybean products also have significant nutraceutical value. Astawan et al. described that tempeh flour revealed higher cholesterol-binding and lipase inhibitory activities than conventional soybean flour, demonstrating its hypolipidemic potential. Further, metabolomics profiling exhibited increased levels of GABA, isoflavones, organic acids, amino acids, and meglutol compounds, responsible for inhibition of cholesterol synthesis and promoting excretion of cholesterol and bile acid. These findings support the use of fermented soybean products in the prevention of dyslipidemia and management of cardiovascular disease.

In another study, Peng et al. demonstrated the anti-obesity efficacy of vinegar prepared from fermented *Camellia sinensis* var. Assamica flowers in high-fat diet-induced obese mice. The fermented vinegar ameliorated glucose and lipid metabolism, reduced hepatic steatosis and oxidative stress, and altered gut microbiota composition significantly with an increase in *Bacteroidota* abundance and reduction in the *Firmicutes*/*Bacteroidota* ratio. The increase in beneficial bacterial families such as *Oscillospiraceae* and *Eubacteriaceae* and the decrease in *Erysipelotrichaceae* indicated improved microbial balance and metabolic homeostasis, favoring its application as a microbiota-targeted anti-obesity functional drink.

Metabolic alterations during the fermentation of traditional cereals have also been studied in depth. Kgoale et al. examined the dynamics of amino acids and phenolic compounds during the fermentation process of amahewu, a fermented maize beverage from Southern Africa, prepared using yellow (YM) and white maize (WM) varieties. Fermentation caused significant metabolic alterations, including reduction of proline and elevated levels of threonine, tyrosine, aspartic acid, and valine due to microbial metabolism. Diverse phenolic transformations were observed, including the appearance of avenanthramides, stable apigenin levels, and decreased caffeic acid in all fermented samples (FWM and FYM). Principal metabolic routes shifted toward isoquinoline alkaloid biosynthesis in white maize, and alanine/aspartate/glutamate metabolism in yellow maize beverages. Overall, amahewu prepared from white maize showed increased metabolic adaptation and fermentation effectiveness compared to yellow maize types, indicating enhanced nutritional value and biochemical consistency.

Recent advances in multi-omics analyses have widened the role of probiotics in metabolic and endocrine disorders. Xu et al. investigated the beneficial effects of *Lactiplantibacillus plantarum* NKK20 in a mouse model of polycystic ovary syndrome (PCOS) through improvements in inflammatory cytokine levels, fasting glucose levels, lipid metabolism, and hyperandrogenism. The probiotic also restored the normal ovarian structure and strengthened the intestinal barrier by increasing ZO-1 and occludin expression, boosting SCFA production, and lowering endotoxin levels. Microbial analysis further revealed beneficial taxa progression, such as *Bacteroidetes* and *Muribaculum*, while metabolomics analysis confirmed the regulation of steroid hormone biosynthesis and glycerophospholipid metabolism, supporting the therapeutic relevance of microbiome-based strategies in PCOS management.

Fermentation is known to improve bread digestibility and gut tolerance. Ribet et al. emphasized that bread fermentation improves nutrient availability and alleviates gastrointestinal issues related to cereal-based triggers such as Amylase-Trypsin Inhibitors (ATIs), gluten, and fermentable oligosaccharides, disaccharides, monosaccharides, and polyols (FODMAPs). Simultaneously, fermentation fosters the creation of advantageous metabolites like exopolysaccharides, SCFAs, and mineral bioaccessibility, thus supporting gut health and overall digestibility.

Nevertheless, fermented dairy products are also one of the most extensively studied probiotic foods, which are known for their health benefits. Arslan et al. reviewed the beneficial effects of yogurt, sour cream, kefir, cheese, and buttermilk on gastrointestinal health, immune regulation, metabolic balance, and cardiovascular function. The beneficial effects are attributed to modulation of the gut microbiota, stabilization of epithelial barrier integrity, and production of bioactive metabolites such as exopolysaccharides, organic acids, peptides, and postbiotic compounds. In their second review, Agagündüz et al. introduced the concept of “multibiotics,” which includes probiotics, prebiotics, postbiotics, and multifunctional metabolites (“multimetabolites”) generated during dairy fermentation. Although fermented dairy products demonstrate substantial therapeutic promise, translational challenges persist due to variability in microbial strains, fermentation conditions, product composition, and host-specific responses. Therefore, standardization of fermentation methodologies and rigorous clinical validation are essential for translating these functional foods into evidence-based nutritional interventions.

In summary, the articles published in this Research Topic demonstrate that fermented foods are not only nutrient-rich but also function as active systems influencing gut microbiota, metabolism, and overall host physiological processes. Integrating microbiome science, metabolomics, fermentation engineering, and systems biology enables the development of targeted functional foods and personalized nutrition strategies to prevent and manage inflammatory, metabolic, and digestive disorders.

